# The Mozart Effect on the Episodic Memory of Healthy Adults Is Null, but Low-Functioning Older Adults May Be an Exception

**DOI:** 10.3389/fpsyg.2020.538194

**Published:** 2020-11-03

**Authors:** Susana Silva, Filipa Belim, São Luís Castro

**Affiliations:** Center for Psychology at University of Porto, Faculty of Psychology and Education Sciences, University of Porto, Porto, Portugal

**Keywords:** music listening, Mozart effect, episodic memory, preference, aging

## Abstract

Literature on the effects of passive music listening on cognitive performance is mixed, showing negative, null or positive results depending on cognitive domain, age group, temporal relation between music and task (background music vs. music before task, the latter known as Mozart effect), or listener-dependent variables such as musical preference. Positive effects of background music on the two components of episodic memory – item and source memory - for verbal materials seem robust and age-independent, and thus deserve further attention. In the current study, we investigated two potential enhancers of music effects on episodic memory: stopping music before task performance (Mozart effect) to eliminate music-related distraction and using preferred music to maximize reward. We ran a main study on a sample of 51 healthy younger adults, along with a pilot study with 12 older adults, divided into low- vs. high functioning according to cognitive performance in a screening test. Against our expectations, Bayesian analyses showed strong evidence that music had no advantage over silence or environmental sounds in younger adults. Preferred music had no advantage either, consistent with the possibility that music-related reward had no impact on episodic memory. Among older adults, low- but not high-functioning participants’ item memory was improved by music – especially by non-preferred music - compared to silence. Our findings suggest that, in healthy adults, prior-to-task music may be less effective than background music in episodic memory enhancement despite decreased distraction, possibly because reward becomes irrelevant when music is stopped before the task begins. Our pilot findings on older adults raise the hypothesis that low-functioning older participants relate to prior-to-task auditory stimulation in deviant ways when it comes to episodic memory enhancement.

## Introduction

Music is incorporated in many aspects of everyone’s life. The act of listening to music is often motivated by the search for aesthetic experiences or affective regulation (Groarke and Hogan, 2018), but there is also an increasing awareness that passive music listening – i.e., listening without analytical intentions – may enhance cognitive performance (for cognitive effects of music training, see Kraus and Chandrasekaran, 2010; Besson et al., 2011). Along with research on younger adults, attention has been paid to the benefits that passive music listening may have for older adults, including those with cognitive impairments (Thaut, 2010; Peck et al., 2016; Fang et al., 2017. Determining when and how passive music listening enhances cognitive performance in younger adults is an important practical goal, in that it may provide directions for the optimization of their study/work conditions. Doing the same for older age groups is perhaps an even more critical goal: as the world tries to deal with the consequences of increased longevity, cognitive improvement in older ages becomes a priority.

Despite the potential that lies in promoting cognitive enhancement through passive music listening, research findings remain mixed. These findings fall into three main research topics: sung vs. spoken words, background music effects, and neuropsychological priming via music (related to the Mozart effect tradition, see below). In all these topics, we find evidence of negative, null and positive impact of music across different cognitive domains and populations.

Research on *sung vs. spoken words* evaluates the impact of presenting sung versions of verbal material to be encoded and later recalled or recognized. Studies with younger adults have pointed to disadvantages (Racette and Peretz, 2007) or limited advantages (Wallace, 1994) of sung words over regular speech in subsequent word recall. In healthy older adults, the impact of singing words during encoding on subsequent verbal recognition seems null (Simmons-Stern et al., 2010). In contrast, Alzheimer’s disease patients seem to benefit from listening to sung words for later recognition (Simmons-Stern et al., 2010) and recall of verbal material (Moussard et al., 2012).

A second research topic relates to *background music*, i.e., the presence of music during task performance. For younger participants, negative effects of music on reading, memory (Cassidy and MacDonald, 2007; Kämpfe et al., 2011; Thompson et al., 2012; Yang et al., 2015) and complex tasks (Gonzalez and Aiello, 2019) have been widely reported. These have been associated to distraction, dual-tasking and cognitive load. An extreme scenario of detrimental music-effects on younger adults may be found in studies using the Irrelevant Sound Effect paradigm (Perham and Vizard, 2011), in which the task to be accomplished requires serial ordering and thus may conflict with the ordinal information (sequence of events) of background music itself. Null effects of background music on working memory have been reported for younger adults (Chew et al., 2016), and positive effects on various domains may be found for both age groups: effects on foreign language learning (Kang and Williamson, 2014, younger adults), category fluency (Thompson et al., 2005, older and Alzheimer’s disease patients), working memory (Mammarella et al., 2007; Mammarella, 2017, older adults), autobiographical memory (Irish et al., 2006, Alzheimer’s disease; Foster and Valentine, 2001, mild and moderate dementia), semantic memory, processing speed (Bottiroli et al., 2014, older adults), verbal encoding (Ferreri et al., 2013, younger adults) and episodic memory (Bottiroli et al., 2014; Ferreri et al., 2014, 2015, older and younger adults).

Finally, a third approach tests the effects of listening to music prior to task performance, in the tradition of the so-called *Mozart effect* (Rauscher et al., 1993) and in line with the idea of neuropsychological priming (Cassidy and MacDonald, 2007). Again, we find mixed results: negative effects on working memory (Giannouli et al., 2019, younger and older adults), null effects on working memory training (Borella et al., 2019, older adults) and attention (Lake and Goldstein, 2011, mild cognitive impairment patients), as well as positive effects on word fluency (Giannouli et al., 2019, younger adults) and image encoding (Carr and Rickard, 2016, younger adults). Positive effects of music – whether as embedding sung words, as background, or as prime – have been related to the idea that music may be a source of mood improvement, arousal, or reward (Blood and Zatorre, 2001; Schellenberg, 2005; Salimpoor et al., 2013; Ferreri and Verga, 2016). While mood improvement tends to enhance cognitive performance due to increased dopamine levels in the brain (Ashby et al., 1999), arousal is known to act according to an inverted-U shape, where both extremely low and extremely high arousal damage performance while moderate levels benefit it (see Thompson et al., 2001, for a review). As for reward, the key to cognitive improvement seems to lie in the role of mesolimbic reward system in memory formation during reward-motivated learning (see Ferreri and Verga, 2016).

When facing this heavily mixed picture, identifying patterns of effectiveness is crucial, namely those concerning the cognitive domains that seem to respond most sensitively to music stimulation. Among reports of positive music-effects, studies on episodic memory for verbal materials (Ferreri et al., 2013, 2014, 2015; Bottiroli et al., 2014) stand out for their consistency: positive effects were replicated, and they were found both in healthy younger (Ferreri et al., 2015) and older adults (Bottiroli et al., 2014; Ferreri et al., 2014). Moreover, these studies showed that background music facilitates the encoding of printed verbal materials not only when music is compared to a silent context, but also when compared to non-musical auditory contexts such as environmental sounds or noise. This suggests that the impact of music on episodic memory is specific, rather than reflecting a general advantage of sound over silence. Episodic memory for verbal materials seems, thus, like a promising domain to test when it comes to considering the potential benefits of passive music listening. However, positive music-effects on episodic memory for verbal materials have been investigated only from the perspective of background music. Therefore, an important question is whether such positive effects extend to priming paradigms, i.e., when music is presented before the memory task and participants perform in silence. Both background and priming paradigms are thought to enhance arousal, reward and mood (Blood and Zatorre, 2001; Schellenberg, 2005; Salimpoor et al., 2013; Ferreri and Verga, 2016), but priming (non-task-concurrent music) is potentially less detrimental in terms of dual-task, cognitive load and distraction (Borella et al., 2019). From this viewpoint, it is possible that positive effects from music on episodic memory become more visible under priming than under concurrent stimulation: priming could keep the benefits of music, while minimizing its costs.

Another pattern that can be found in the literature concerns the moderating role of musical preference – the extent to which listeners enjoy the music style that is used as background or prime – in boosting the positive effects of music. Preference does not seem to change the Irrelevant Sound Effect (Perham and Vizard, 2011), but – at least in younger adults – it boosts the positive effects of background music on reading comprehension (Mcdonald, 2013), as well as those of musical primes on image encoding (Carr and Rickard, 2016): in both cases, preferred music outperforms silence, while non-preferred music does not. One explanation for the advantage of preferred music is that it is rewarding (Blood and Zatorre, 2001; Ferreri and Verga, 2016), and reward may be one mechanism subtending the positive effects of music on cognition (Ferreri and Verga, 2016). Despite the possibility that preference is a boosting factor because it maximizes reward, its moderating role has not been considered while approaching the impact of music on episodic memory.

In the present study, we investigated the impact of prior-to-task music on episodic memory in a sample of 51 healthy younger adults (Experiment 1) and we examined how preference modulates this impact. Following Ferreri et al. (2015)’s paradigm, we manipulated the auditory context associated to encoding printed words: silence, environmental sounds, musical excerpts. Unlike Ferreri et al. (2015), who presented the auditory context 20 s before the task began and kept it going as background while the task unfolded, we turned off the sound as the task began (prior-to-task stimulation). For comparison with Ferreri et al. (2015), we used the same pre-task 20-sec stimulation period, even though experiments on the Mozart effect tend to use longer stimulation times (a few minutes, see, e.g., Rauscher et al., 1993). We were aware that this could limit the impact of music but, in the absence of evidence regarding minimum auditory stimulation times to grant the Mozart effect, we moved on with our choice, which would grant maximum comparability with Ferreri et al.’s (2015) settings. We then tested auditory context effects on later recognition considering the two components of episodic memory: item memory and source memory (Easterbrook, 1959; Glisky et al., 1995). While item memory stands for the content of the encoding context, source memory represents the context in which the content is being encoded. In Ferreri et al.’s (2015) study, music had an advantage over environmental sounds and silence in both item (recognizing a printed word as old or new) and source memory (indicating the auditory context where the word appeared), suggesting that music is beneficial for episodic memory in its two dimensions. To grant the presence of a source component – an encoding context – in our study, we presented the auditory context not only before, but also after task performance: by doing this, we prevented the association of words to the following auditory context. To investigate the moderating role of preference, we then asked participants to rate their preference for the three musical excerpts, and we compared silence, environmental sounds, preferred and non-preferred music. Based on the idea that non-task-concurrent music (prime) could keep the benefits of background music (arousal, reward or mood improvement) while minimizing its distraction-related costs, we predicted that music would have strong advantage over silence and/or environmental sounds. Concerning preference, we predicted an advantage of preferred over non-preferred music, based on previous findings (Mcdonald, 2013; Carr and Rickard, 2016) as well as on the principle that preference could maximize the reward component of music.

The literature on episodic memory suggests that background music may have similar positive effects on younger and healthy older adults (Bottiroli et al., 2014; Ferreri et al., 2014, 2015). However, there is also evidence that, among older adults, those with cognitive impairments may be more responsive to music effects on cognition due to impairment-related mechanisms that add to the general mechanisms of arousal, reward or mood improvement: on the one hand, music may recruit brain areas spared after degeneration in cognitively impaired individuals and elicit compensatory mechanisms in the brain (Ferreri and Verga, 2016) which are not activated in healthy participants under the same music stimulation; on the other hand, music may act by reducing task-related anxiety, which is expected to be higher in cognitively impaired participants (Irish et al., 2006). For instance, Simmons-Stern et al. (2010) found benefits in sung (vs. spoken) words in Alzheimer’s patients, but not in younger healthy participants. In order to make a preliminary approach to the possibility that low-functioning older adults could show increased sensitivity to the Mozart effect on episodic memory, we ran a second, pilot, experiment (Experiment 2), with a small group of older adults, split into low- vs. high-functioning concerning cognitive status.

Comparisons between the three groups – younger, high-functioning older and low-functioning older adults - were complemented with the analysis of sensitivity to possible semantic associations between music and words and to possible serial order effects (primacy/recency) on item memory.

## Experiment 1

### Methods

#### Participants

Fifty-one undergraduate Psychology students (45 women, mean age ± *SD* = 19.9 ± 1.9 years) participated in the experiment. All participants had normal hearing and normal or corrected-to-normal vision, and did not report any psychiatric, neurological or cognitive problems. All signed informed consent according to the Declaration of Helsinki.

#### Stimulus Materials

Verbal stimuli consisted of 45 + 45 = 90 words selected from the PORLEX database (Gomes and Castro, 2003, see Supplementary Appendix A). One set of 45 words was presented at the encoding phase (old words, to be remembered), and both sets (45 old + 45 new) were presented at the test phase. Old and new words were matched for length, frequency and morphological status (verb, noun or adjective).

Audio stimuli (auditory contexts before and after encoding) consisted of five 20-s audio files containing silence, environmental sounds (water running and birds, simultaneously) and three instrumental (non-vocal) music excerpts. We chose instrumental music because it may be less detrimental to performance in verbal tasks than vocal music (Crawford and Strapp, 1994). The audio file containing environmental sounds was extracted from a recording that was available online^1^.

The three music excerpts were selected from an initial pool of 12 instrumental pieces, following an online pre-test with 20 university students (see Supplementary Appendix B). The pre-test was run with the goal of selecting maximally contrasting music stimuli in terms of preference. The initial set contained excerpts from four different and potentially contrasting music genres (3 examples per genre, 3 × 4 = 12): metal, hip hop, electronic and jazz. For each music excerpt, participants were asked to rate their level of preference (scale with 10 levels, where 1 means “I don’t like it” and 10 “I like it a lot”). We analyzed the pre-test data per subject, with the initial aim of determining preference contrasts: we listed the most contrasting pairs of excerpts per subject and then counted the frequency of occurrence of such pairs across subjects. The final selection included “John and the Creatures – Here’s to the Crazy Ones” (metal genre), “Robert Miles – Children” (electronic genre) and (“Thelonious Monk - Blue Monk”) from jazz genre (Supplementary Appendix B).

All audio files except the silent one were normalized to 70 dB. The start and end points of music excerpts coincided with structural breaks in the musical piece, thus avoiding abrupt transitions.

#### Procedure

For the encoding phase, the 45 old words were randomly divided into five lists of nine words (5 × 9 = 45), and each of these five word-lists was presented in a different auditory context (silence, environmental sounds, music 1/metal, music 2/electronic, music 3/jazz).

We created three versions of the encoding phase, each with a different pairing between word lists and auditory context. We did this by keeping the order of words constant while switching the order of auditory contexts: silence-environmental sounds-music vs. environmental sounds-music-silence vs. music-silence-environmental sounds. The three versions of the experiment were balanced across subjects. The goal of having multiple versions of the experiment - each with its own pairing between words and auditory contexts - was to control for two types of confounds that could act upon the auditory context effects we were interested in. One confound related to possible pre-existing semantic associations between a specific word and a given auditory context: for instance, the word “alumínio” [aluminum] could be associated to the metal music genre more easily than to silence or nature sounds (see Ferreri and Verga, 2016) due to the word’s relation with the name of the genre (metal). If all participants saw the word coupled with metal music, they could have better encoding of both the word (item memory) and the auditory context (source memory) due to the semantic association, thus falsely increasing the advantage of music contexts. Different pairings between words and auditory contexts, distributed across participants, would counteract this type of confound. The other confound we wanted to control for concerned primacy/recency effects on item memory, i.e., the possibility of words at the top/bottom of the list being more memorable and lending an advantage to the first/last context presented. Again, while a single pairing between words and auditory contexts could lend a spurious advantage to words coupled with the first (primacy) or last context (recency), different versions of the experiment would dilute this influence.

The 20-s auditory context (music, environmental sounds or silence) was presented before and after each word list. Single words were presented on the screen for 5 s, always preceded by a 200-ms fixation cross (46 800 ms for each nine-word list). Thus, participants started each of the five blocks of the experiment by listening to the auditory context for 20 s, then they saw the words in silence (46.8 s) and, finally, they listened again to the auditory context. At the end of each block (auditory context + word list + auditory context), they saw an instruction on the monitor to use the space bar of the computer keyboard to move on to the next block. Participants were instructed to read the words silently and try to memorize as many as they could, since they would be later tested on this. They were not instructed to pay attention to the association between words and auditory context.

At the end of the encoding phase, participants were given a visual discrimination task (XO letter comparison task, Salthouse et al., 1997), lasting 5 min and working as an interference task. This interference task was critical for testing long-term episodic memory performance and not just working memory (Ferreri et al., 2015). The task consisted of examining a pair of letters (including only X and/or O) and deciding as quickly as possible whether the letters were the same (e.g., XX) or different (e.g., XO). Participants responded by pressing one of two keys in the computer keyboard.

After the interference task, participants were tested for discrimination between old and new words (item memory), as well as for their memory of the auditory context in which the word was presented (source memory). Thus, in this test phase, participants saw each of the 45 old + 45 new words, presented in pseudorandomized order: first, we did a full randomization using algorithmic procedures, and then we adjusted the list in order to avoid patterns concerning the alternation between old and new. They were then asked two different questions: first, “did you see this word before? Yes or No?”, and second, “In which circumstances did you see it?”. Here, there were four response options – Silence, Environmental sounds, Music or Did not see it before. Participants responded Yes or No by pressing either the left or the right Control (Ctrl) key in the computer keyboard. Half the participants saw “Yes No” on the monitor and were instructed to use the left Ctrl key for Yes. The other half saw “No Yes” and used the right one for Yes. As for the second question, the options were numbered as 1, 2, 3, 4, respectively, and participants pressed the corresponding number-key.

At the end of the experimental session, participants listened again to the three music excerpts and were asked to rate each of these for preference (1 to 10). These ratings allowed us to determine the contexts of preferred-music and non-preferred-music for each participant. The experiment was performed in a quiet room, and stimuli were delivered using Presentation software^2^. We used a 15-inch monitor for visual display and high-quality headphones for audio reproduction. Each session lasted approximately 40 min.

#### Analysis

Preference ratings were first tested for significant contrasts across musical excerpts, to validate the idea that we were dealing with different levels of preference.

To determine the effects of auditory context, subject-level d-prime values (Stanislaw and Todorov, 1999) were computed for each of the five contexts (*silence, environmental sounds, metal, electronic, and jazz music*). D-prime is a measure of discrimination, and it is based on differences between hit rates (correct classification of the target) and false alarms (classification of non-targets as targets). A value of zero indicates no discrimination, and negative values point to reversed classifications (non-targets dominantly classified as targets and vice-versa). Values were calculated for item memory (discrimination between old and new verbal items) and source memory (discrimination between the target auditory context and the other ones). By averaging d-prime values for the three musical excerpts, we obtained d-prime for *music*. Based on each participant’s preference ratings, another set of d-prime values were calculated: for the music context(s) with highest preference (*d-prime preferred music*), for the one(s) with lowest preference (*d-prime non-preferred music*). In some cases, participants rated a single excerpt as the most liked (e.g., score of 9 against 7 and 5) and a single excerpt as the least liked (the one with a score of 5, in the previous example). In other cases, two of the excerpts had the same score (e.g., 9, 9, 5): in these cases, we averaged the d-prime values of those two excerpts in order to define the d-prime (for preferred music, in this example).

Effects from auditory context were tested three times: first, comparing silence, environmental sounds and music (*general auditory context*); second, comparing silence, environmental sounds, preferred and non-preferred music (*preference-related auditory context*); finally, considering the five *specific auditory contexts* (silence, environmental sounds, metal music, electronic music, jazz music). For each analysis, we considered the effects on both item memory (discrimination between old and new words as a function of auditory context) and source memory (discriminant identification of contexts). All within-subject comparisons were made with repeated measures ANOVAs, using auditory context as factor and d-prime as dependent variable.

We made a complementary mixed ANOVA using *experiment version* (silence-environmental sounds-music vs. environmental sounds-music-silence vs. music-silence-environmental sounds) as between-subjects factor and general auditory context (silence, environmental sounds, music) as within-subjects. As we mentioned in the Procedure section (see above), experiment version manipulations were made to dilute potential confounds from *semantic association* or *serial order effects*, and thus they were not a critical part of our research question. However, examining effects from experiment version could allow us to identify such confounds, contributing to future improvements of the current paradigm and expanding the comparative analysis of younger vs. older adults’ susceptibility to these. The use of *extended semantic associations* between words and auditory contexts by participants would be indicated by main effects of experiment version on both item and source memory – meaning that a particular experiment version carried semantic associations between the globality of word lists and their respective auditory contexts (e.g., version where the word ‘petrify’ was paired with silence, ‘aluminum’ with metal music and ‘seagull’ with environmental sounds), favoring both item and source recognition in that particular version. The use of *local semantic associations* would be indexed by interactions between experiment version and auditory context on both item and source memory, meaning that one and only one particular word list could be perceived as semantically related to its encoding context (e.g., a word list containing ‘petrify’ paired with silence in a given version, without pairings between ‘aluminum’ with metal music or ‘seagull’ with environmental sounds), favoring silence as an encoding context in that version. As for *serial order effects*, they would be manifested by interactions between experiment version and auditory context on item memory: if an auditory context elicited higher levels of performance in item memory when it appeared early in the experiment this could index *primacy* effects; in the reverse case (auditory context favoring item memory only when appearing late in the experiment), *recency* effects could be considered.

Unless otherwise specified, the adopted critical level of significance was 0.05. Violations of sphericity were compensated with Greenhouse-Geisser corrections. When significant effects from auditory context were observed, we carried out pairwise comparisons across the different levels of the factor using Bonferroni corrections for multiple comparisons. For the mixed ANOVA, significant interactions were broken down into auditory context effects per experiment version. The null-hypothesis-significance-testing procedure was complemented with the estimation of Bayes factors for null results, as delivered by JASP (JASP Team, 2020). Bayes factors for null results [null/alternative]) indicate the likelihood of observing the null data under the null hypothesis compared to the alternative hypothesis, thus quantifying the strength of evidence in favor of absent effects (Hoekstra et al., 2018; Wagenmakers et al., 2018). A rule of thumb is to consider a Bayes factor between 1 and 3 as weak evidence, though favoring the null hypothesis, between 3 and 10 as substantial, between 10 and 30 strong, and between 30 and 100 as decisive evidence (Jarosz and Wiley, 2014). Bayes factors can also be used to estimate the likelihood of the data under the alternative hypothesis compared to the null one (Bayes factor [alternative/null].

### Results

#### Preference Ratings

Average preference ratings were 5.12 (*SD* = 2.48) for the metal excerpt, 3.86 for electronic music (*SD* = 1.97) and 5.76 for jazz (*SD* = 2.13). Average rating contrasts were 1.25 for metal vs. electronic (*SD* = 2.62), 1.90 for jazz vs. electronic (*SD* = 2.66) and 0.65 for jazz vs. metal (*SD* = 3.03). Ratings of electronic music were significantly lower than ratings of metal (*t*(50) = 3.40, *p* = 0.001, *d* = 0.57) and jazz (*t*(50) = −5.10, *p* < 0.001, *d* = 0.89). Metal and jazz excerpts did not differ from each other (*p* = 0.13).

#### Effects of General Auditory Context

General auditory context (silence vs. environmental sounds vs. music) had no effects on item memory (*p* = 0.63, η^2^p = 0.009) and source memory (*p* = 0.51, η^2^p = 0.013). Bayes factors (null/alternative) indicated that data for item memory and source memory were 10.39 and 8.61 times more likely to occur under the null hypothesis than under the alternative one, showing strong and substantial evidence for the null hypothesis, respectively (Jarosz and Wiley, 2014).

#### Effects of Preference-Related Auditory Context

Comparisons between silence, environmental sounds, preferred music and non-preferred music did not reveal significant effects, neither on item memory (*p* = 0.67, η^2^p = 0.010) nor on source memory (*p* = 0.88, η^2^p = 0.004). Bayes factors (null/alternative) of 21.86 for item memory and 31.29 for source memory indicated strong and very strong support to the null hypothesis, respectively.

#### Effects of Specific Auditory Context

**FIGURE 1:**
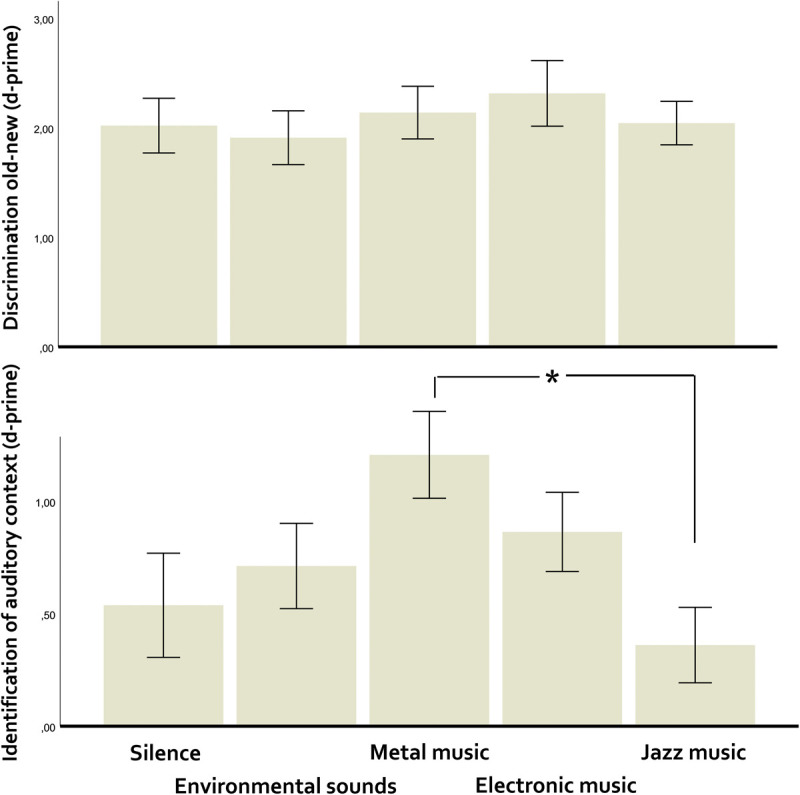
Discrimination old-new (item memory, above) and discriminant identification of all auditory contexts (source memory, below) as a function of specific auditory context (including contrasts across music genres). Vertical bars represent the standard error of the mean. The asterisk indicates significant differences.

When comparing silence, environmental sounds and each of the three music excerpts, effects on item memory were null (*p* = 0.68, η^2^p = 0.011: Bayes factor, BF (null/alternative) = 39.10), and those on source memory were significant (*F*(4,200) = 3.72, *p* = 0.006, η^2^p = 0.069, BF [alternative/null] = 4.06, Figure 1). Metal music was significantly better identified than jazz as the auditory context of old words (*p* = 0.003, *d* = 0.67).

#### Semantic Associations and Serial Order Effects as Revealed by Experiment Version

**FIGURE 2:**
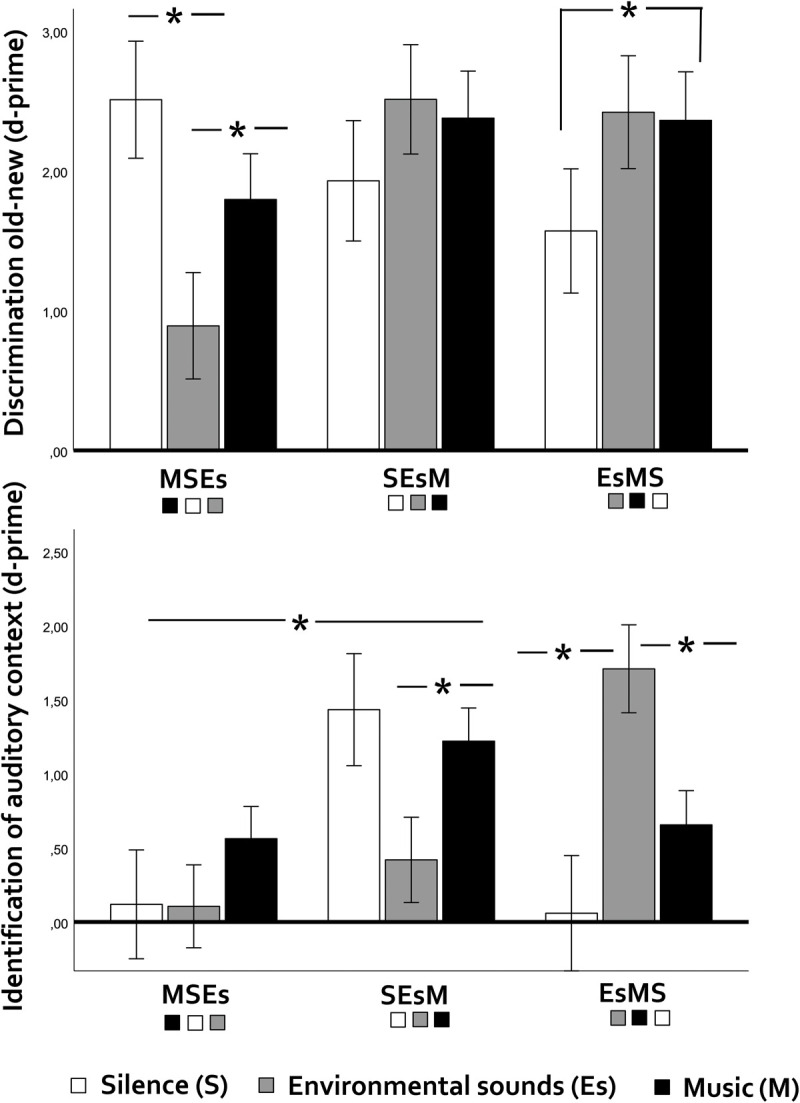
Discrimination old-new (item memory, above) and discriminant identification of preference-related auditory contexts (source memory, below) in younger adults as a function of general auditory context (silence-environmental sounds, music) and experiment version (three different orderings of silence, environmental sounds and music blocks). Vertical bars indicate the standard error of the mean. Asterisks indicate significant differences. Note that – when contexts differ significantly, the last context presented in each version elicits the worst performance in item memory, indicating primacy effects. Asterisks indicate significant differences.

There was no evidence that semantic associations between words and auditory contexts were being exploited by participants: Main effects of experiment version (silence, environmental sounds vs. music; environmental sounds, music, silence vs. music, silence, environmental sounds) – which could indicate *extended* semantic associations if present for both memory types – were significant for source memory (*F*(2,48) = 3.39, *p* = 0.042, η^2^p = 0.124, Figure 2) but null for item memory (*p* = 0.41, η^2^p = 0.036; BF[null/alternative] = 1.04). In source memory, participants who completed the silence – environmental sounds (Es) – music version outperformed those who listened first to music, then silence, and then Es (*p* = 0.045, *d* = 0.74). Please note that, although the Bayes factor for the null data in item memory was weak (1.04), the inverted Bayes factors (alternative/null) differed substantially for item (BF = 0.957) vs. source memory (BF = 66.72).

As for the interaction between experiment version and auditory context, it was significant for both item (*F*(4,96) = 5.14, *p* = 0.001, η^2^p = 0.177, BF [alternative/null] = 3.74) and source memory (*F*(4,96) = 7.06, *p* < 0.001, η^2^p = 0.227, BF [alternative/null] = 178.66, Figure 2). At first sight, this could point to *local* semantic associations – i.e., the possibility that one specific word list fits semantically with a specific auditory context, favoring subsequent recognition of words and contexts in the experiment version where this particular combination occurred. However, the interactions showed different patterns for the two types of memory (see Figure 2): in the music-silence-Es version, words presented under silence and music were better recognized than those presented in the Es context (item memory, Es vs. silence: *p* = 0.016, *d* = 1.01; Es vs. music: *p* = 0.016, *d* = 0.80), while auditory context had no effect on source memory. In the Es-music-silence version, music outperformed silence (*p* = 0.012, *d* = 1.08) in item memory, while environmental sounds outperformed both music (*p* = 0.005, *d* = 1.08) and silence (*p* < 0.001, *d* = 1.46) in source memory. In the silence-Es-music version, auditory context had no effects on item memory, while music outperformed Es in source memory (*p* = 0.001, *d* = 0.60).

Although local semantic associations do not seem to account for the interactions between auditory context and experiment version on item vs. source memory, *primacy-related effects* (expected for item memory only) are consistent with the observed pattern of interactions: whenever there were context effects on item memory, the last context (last word list) always elicited the worst performance (see Figure 2). Therefore, there seems to be evidence that recency was detrimental to performance in younger adults.

### Discussion

Unlike our prediction, music contexts did not enhance episodic memory compared to silence or environmental sounds, suggesting that the advantage of background music that had been seen in previous studies (Bottiroli et al., 2014; Ferreri et al., 2014, 2015) may vanish if music is stopped before task performance. Also unlike our prediction – but consistent with the possibility that music-related reward did not improve episodic memory - music preference had no moderating role.

Additional comparisons, considering specific musical pieces, indicated an advantage of heavy metal over jazz music in source memory. One possibility is that this was due to increased physiological arousal (Rickard, 2004), since the jazz excerpt was lighter in terms of texture and event density compared to heavy metal. From this viewpoint, it is possible that physiological arousal overrided reward in the enhancement of younger adults’ source memory. Given that we did not measure arousal in participants, this is, however, just a hypothesis.

From the analysis of experiment version effects, we found no evidence that younger participants were responding based on semantic associations between words and auditory contexts. However, the interactive effects of experiment version and auditory context on item memory suggested that the most recently presented items may have been encoded more poorly, possibly revealing a primacy effect on item memory, or a decrease in attention levels due to fatigue.

Experiment version had a main effect on source memory: participants had better recognition of the auditory context associated with a given word when auditory contexts were ordered as silence, environmental sounds and music during encoding. As we already pointed out in the Results section, this pattern is not consistent with effects from semantic associations (otherwise it should be present for item memory too). So, what else may account for the positive impact of ordering auditory contexts as silence, environmental sounds and music on source memory? One possibility is that participants were accurate in recency memory – one measure of source memory referring to awareness of the early vs. late appearance of words in the encoding phase (Czernochowski et al., 2008) – but they were not aware of the order of sources (e.g., music appearing early, followed by silence and then by environmental sounds). Under uncertainty, participants might have assumed that contexts were ordered as silence, environmental sounds and music, since this corresponds to a logical order that starts with no information (silence), follows into low-structured information (environmental sounds) and ends with high-structured information (music). Therefore, when confronted with a word they knew had been presented early in the experiment, they might have assumed the word appeared in a silent context; if they thought the word appeared at the end, they would respond ‘music’. When auditory contexts were ordered as silence, environmental sounds and music, this strategy would grant high performance in source memory. In the other two versions, it would not work. In Experiment 2, we looked again into these experiment version effects to see whether and how they act in older adults.

## Experiment 2

This experiment was designed as a pilot study to test the hypothesis that low-functioning older adults may be more sensitive than healthy younger and older adults to music effects. Since we were interested in a homogenous sample in terms of socio-economical and cultural background, we recruited participants from a nursing-home located at a small community. The available sample was already small, and eligibility criteria (see below) made it even smaller (n = 12). Considering the effects of normal aging on cognitive functioning and the low educational levels of our participants (see below), we simplified the stimulus set with the goal of avoiding experimental stress and/or floor effects. In order to understand how the effects of musical context may depend on lower vs. higher cognitive status, we administered the Mini Mental-State Examination (MMSE) adapted to the Portuguese population (Guerreiro et al., 1994).

### Methods

#### Participants

Twelve healthy older adults (7 women, mean age ± *SD* = 75.25 ± 8.3 years; mean schooling ± *SD* = 4.92 ± 2.39 years) from a nursing home participated in the experiment. These participants were selected by the local health technician, based on the absence of incapacitating deficits (able to read, speak, communicate, corrected vision and hearing). Our examination of cognitive functioning using MMSE indicated normal levels of performance in seven participants (high-functioning), and five cases below the cut-off score (low-functioning): 17, 19, 21, 22, 22, cut-off of 24 (Morgado et al., 2009).

Prior to any contact with the participants, the local ethics committee approved the experiment. All participants signed informed consent according to the Declaration of Helsinki.

#### Stimulus Materials

Given that participants had very low educational levels, we reduced substantially the size of the stimulus set. Verbal stimuli consisted of 10 + 10 words (20) words, taken from the list used in Experiment 1 (see Supplementary Appendix C). We selected words with lower frequency and length to facilitate encoding. One set of 10 words was presented at the encoding phase (old words, to be remembered), and both sets (old and new, 10 old + 10 new) were presented at the test phase.

Silence and environmental sounds stimuli were the same as in Experiment 1. We selected music stimuli based on our previous knowledge about the socio-economic, generational and cultural background of participants: we considered two genres likely to be familiar and/or preferred (fado and traditional local music), contrasting with hip-hop – highly likely to be non-preferred. Again, the idea of contrasting musical styles served to prevent the possibility of the same person having the same preference for all pieces in the list. Auditory stimuli were processed in the same way as in Experiment 1.

The Mini Mental State Examination was administered to assess cognitive functioning of each participant. MMSE is one of the most commonly used screening tools and assesses global cognitive functions in clinical or research contexts, and it is suited to individuals with low educational levels (Guerreiro et al., 1994).

#### Procedure

The procedure was similar to Experiment 1, except that there were only 10 old words pseudo-randomly divided into five lists of 2 words each. Each list was presented in-between silence, environmental sounds, music 1/hip hop, music 2/fado music and 3/traditional local music.

Mini Mental State Examination (MMSE) was administered before the experimental task. To minimize difficulties associated to the interaction with the computer, the experimenter pressed the keyboard keys after participants provided their responses vocally.

#### Analysis

We ran the same analyses as in Experiment 1, adding cognitive functioning (low-, below MMSE cut-off score, *n* = 5, vs. high-functioning participants, equal or above cut-off, *n* = 7) as between-subjects factor to all. Given that the two groups were differently distributed across the three versions of the experiment (low-functioning: one participant in version 1, one in version 2, three in version 3 vs. high-functioning: three in version 1, three in version 2, one in version 3), we paid critical attention to experiment version effects when interpreting the effects of cognitive functioning.

Due to the limited size of our sample, and also because cognitive status as dichotomous variable generated imbalanced groups (5 vs. 7), we did a cross-check analysis with MMSE scores as covariate.

### Results

#### Preference Ratings

Average preference ratings for fado, hip-hop and traditional music were 7.5 (*SD* = 1.93), 4.17 (*SD* = 2.55), and 8 (*SD* = 1.95), respectively. Average rating contrasts were 3.33 (*SD* = 2.93) for fado vs. hip-hop, 0.50 (*SD* = 2.84) for fado vs. traditional music and 3.83 (*SD* = 3.61) for hip-hop vs. traditional music. Hip-hop ratings were significantly lower than fado (*t*(11) = 3.93, *p* = 0.002, *d* = 1.49) and traditional music (*t*(11) = 3.67, *p* = 0.004, *d* = 1.70).

#### Effects of General Auditory Context and Cognitive Functioning

**FIGURE 3:**
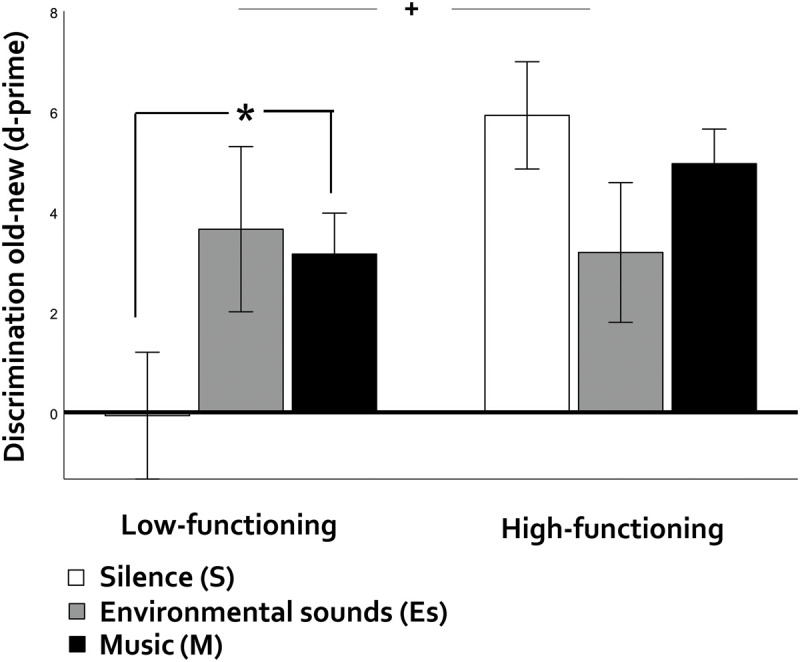
Discrimination old-new (item memory) in older adults as a function of general auditory context (silence-environmental sounds, music) and cognitive functioning (low vs. high). Vertical bars represent the standard error of the mean. The asterisk indicates significant differences and the symbol ‘+’ marginal ones. In the low-functioning group, music, but not environmental sounds outperformed silence.

Cognitive functioning had a marginal main effect on item memory (*F*(1,10) = 3.41, *p* = 0.094, η^2^p = 0.255, BF [alternative/null] = 2.47, Figure 3), with low-functioning participants showing poorer performance than high-functioning ones. Effects of cognitive functioning on source memory did not reach significance (*p* = 0.105, η^2^p = 0.241, BF [null/alternative] = 1.99]).

Main effects of auditory context were non-significant for item (*p* = 0.732, η^2^p = 0.041, BF [null/alternative] = 0.869) or source memory (*p* = 0.741, η^2^p = 0.030, BF [null/alternative] = 4.18). For mean hit rates and d-prime values, please see Supplementary Appendix D). However, the interaction between cognitive functioning and auditory context was significant for item memory (*F*(29,3) = 6.71, *p* = 0.006, η^2^p = 0.402). Among low-functioning participants, music, but not environmental sounds, outperformed silence (music: *p* = 0.009, *d* = 1.43, BF [alternative/null] = 17.34; environmental sounds: *p* = 0.42, *d* = 0.96, BF [alternative/null] = 1.07) while high-functioning participants did not show cross-context differences (*p*s > 0.24, BFs [alternative/null] < 1.39). For source memory, the interaction was non-significant (*p* = 0.387, η^2^p = 0.091, BF [null/alternative] = 4.05). Please note that experiment version did not interact with auditory context (please see below), indicating that the effects of auditory context on low- but not high-functioning participants were not due to group differences in the allocation to different experiment versions.

Cross-check analyses with MMSE scores as covariate showed a significant interaction between auditory context and MMSE on item memory (*F*(2,20) = 5.78, *p* = 0.010, η^2^p = 0.336). Follow-up correlations indicated a strong negative correlation between subject-level advantage of music over silence and MMSE scores (*r*(10) = −0.802, *p* = 0.002), thus reinforcing the idea that music increases its benefits as cognitive status becomes lower. For source memory, the interaction between auditory context and MMSE scores was not significant (*p* = 0.262, η^2^p = 0.125).

#### Effects of Preference-Related Auditory Context and Cognitive Functioning

**FIGURE 4:**
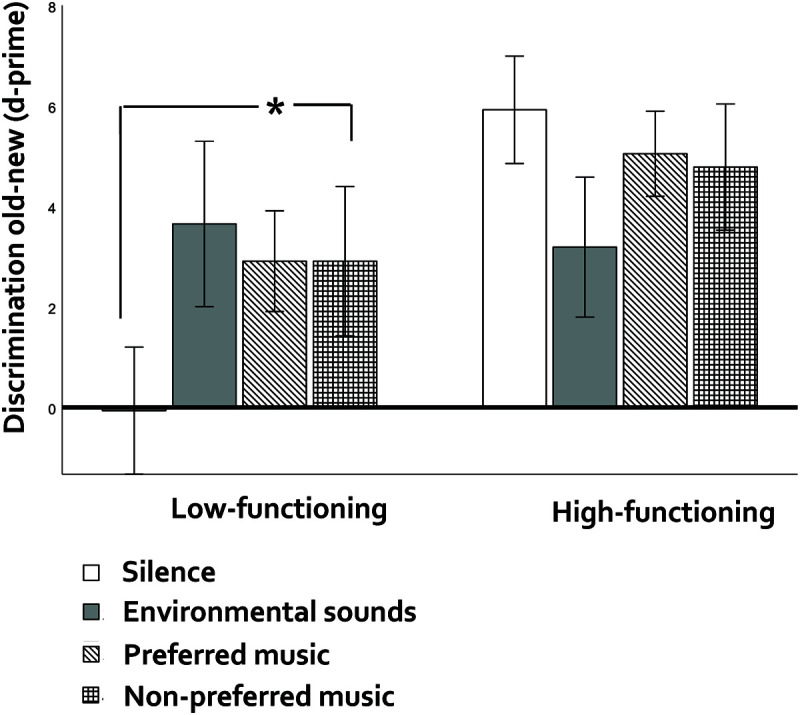
Discrimination old-new (item memory) in older adults as a function of preference-related auditory context (preferred vs. non-preferred music) and cognitive functioning (low vs. high). Vertical bars represent the standard error of the mean. Asterisks indicate significant differences.

Comparisons across silence, environmental sounds, preferred and non-preferred music (Figure 4) showed non-significant results for item (*p* = 0.732, η^2^p = 0.041, BF [null/alternative] = 3.82) and source memory (*p* = 0.701, η^2^p = 0.030, BF [null/alternative] = 7.48).

Cognitive functioning interacted significantly with auditory context for item memory (*F*(1,10) = 3.43, *p* = 0.029, η^2^p = 0.255). Pairwise comparisons for low-functioning participants showed a marginal advantage of non-preferred music over silence (*p* = 0.097, *d* = 1.08, BF [alternative/null] = 4.99 vs. preferred music over silence, BF [alternative/null] = 1.36), while high-functioning participants showed no context effects (*p*s > 0.53, BFs [null/alternative) > 1.05). The cognitive functioning x context interaction was non-significant for source memory (*p* = 0.665, η^2^p = 0.050, BF [null/alternative] = 10.99).

Cross-check analyses with MMSE as covariate confirmed these results, showing a significant interaction between auditory context and MMSE (*F*(1,10) = 3.86, *p* = 0.019, η^2^p = 0.279). Follow-up comparisons showed a marginal negative correlation between the advantage of non-preferred music over silence and MMSE scores (*r*(12) = -0.544, *p* = 0.067). For source memory, there was no significant interaction (*p* = 0.295, η^2^p = 0.144).

#### Effects of Specific Auditory Context and Cognitive Functioning

**FIGURE 5:**
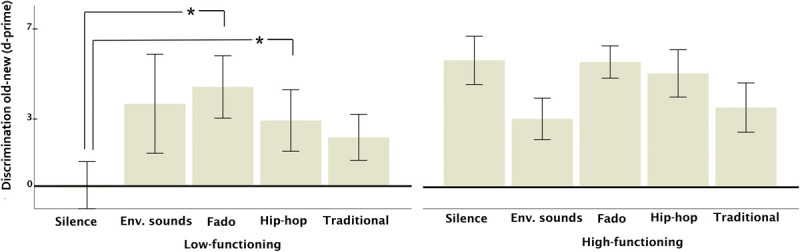
Discrimination old-new (item memory) in older adults as a function of specific auditory context and cognitive functioning (low vs. high). Vertical bars represent the standard error of the mean. Asterisks indicate significant differences.

Comparisons across silence, environmental sounds, fado, hip-hop and traditional music (Figure 5) showed non-significant effects on item (*p* = 0.161, η^2^p = 0.148, BF [null/alternative] = 1.80) or source memory (*p* = 0.489, η^2^p = 0.080, BF [null/alternative] = 5.35), but the interaction between cognitive functioning and context on item memory was significant (*F*(4,40) = 2.84, *p* = 0.036, η^2^p = 0.221, BF [alternative/null] = 1.32). In the low-functioning group, silence showed a significant disadvantage regarding fado (*p* = 0.033, *d* = 1.75) and hip-hop (*p* = 0.016, *d* = 1.18). In the high-functioning group, pairwise comparisons showed no significant differences (*p*s > 0.089).

The interaction between auditory context and cognitive functioning was non-significant for source memory (*p* = 0.762, η^2^p = 0.044, BF [null/alternative] = 11.12).

#### Semantic Associations and Serial Order Effects as Revealed by Experiment Version

**FIGURE 6:**
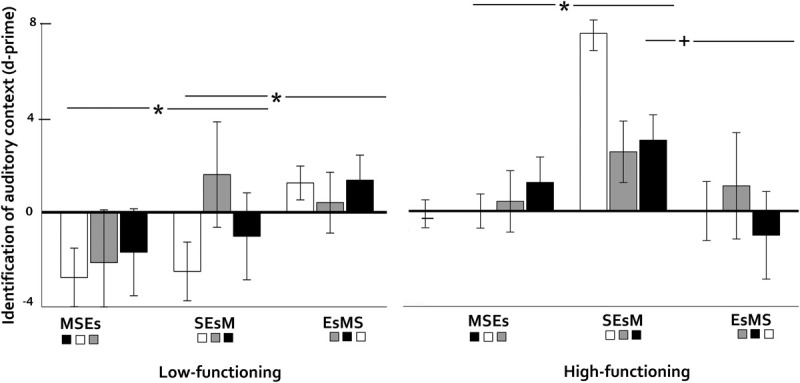
Discriminant identification of general auditory context (silence-environmental sounds, music) in older adults as a function of cognitive status (low – vs. high functioning) and experiment version (three different orderings of silence, environmental sounds and music blocks). Vertical bars represent the standard error of the mean. Asterisks indicate significant differences and the symbol ‘+’ marginal ones.

As in younger adults, there was no evidence of extended (main effect of experiment version) or local (interaction between experiment version and auditory context) semantic associations between words and auditory contexts, which should be reflected into experiment version effects on both item and source memory: Main effects of experiment version were null (*p* = 0.746, η^2^p = 0.093, BF [null/alternative] = 3.06) for item memory with no interactions with cognitive functioning (*p* = 0.593, η^2^p = 0.194, BF [null/alternative] = 2.15), but they were significant for source memory (*F*(2,6) = 8.15, *p* = 0.019, η^2^p = 0.731, BF [alternative/null] = 1.30) with further interactions with cognitive functioning (*F*(2,6) = 10.36, *p* = 0.011, η^2^p = 0.755, BF [alternative/null] = 1.50, Figure 6). Interactions between experiment version and general auditory context were non-significant for item (*p* = 0.755, η^2^p = 0.136, BF [null/alternative] = 3.99) and source memory (*p* = 0.844, η^2^p = 0.102, BF [null/alternative] = 2.22). For both memory types, there were no significant further interactions with cognitive functioning (item: *p* = 0.234, η^2^p = 0.350, BF [null/alternative] = 2.39; source: *p* = 0.218, η^2^p = 0.359, BF [null/alternative] = 0.782).

The lack of interaction between auditory context and experiment version on item memory (BF [null/alternative] = 3.99) indicates that the serial order (primacy) effects which had been observed for younger participants did not hold for older adults.

As for the main effect of experiment version on source memory only (see above, [*F*(2,6) = 8.15, *p* = 0.019, η^2^p = 0.731]), it seems to replicate what we saw in experiment 1 – a possible confound between the real order of contexts and a logical (but unreal) order, but now we must take into consideration the interaction with cognitive function (see above, *F*(2,6) = 10.36, *p* = 0.011, η^2^p = 0.755). Similar to younger adults, both high and low functioning older participants showed improved source memory when listening to sound-Es-music compared to music-silence-sound (Figure 6; high: *p* = 0.009; low: *p* = 0.007). However, in the low functioning group, the most logical order was outperformed by Es-music-silence (*p* = 0.004). So, once again, low-functioning adults deviated from the general pattern.

### Discussion

Unlike younger and older high-functioning participants, music contexts had a positive impact on item memory of low-functioning older participants, showing an advantage over silence. Interestingly, the advantage of music was not driven by preferred music. Instead, when preference-related context effects were tested, it was non-preferred music that showed an advantage over silence. Consistent with this, the least preferred music genres (fado and hip-hop) showed significantly increased benefits to item memory compared to silence.

The order of auditory sources (experiment version) had again an effect on source memory. In older high-functioning adults, the effect was the same as in younger adults (source memory was maximal when auditory contexts were ordered as silence, environmental sounds and music). In the low-functioning group, the order silence-environmental sounds-music was only the second most effective.

Unlike younger adults, item recency was not detrimental to item memory performance of older adults, both high-and low-functioning. This could indicate that either primacy effects were not active in older adults, or that older adults – unlike younger ones - did not get tired or decrease their levels of attention across task blocks. Since the latter possibility is unlikely, we will favor the first interpretation (primacy effects in younger but nor older participants) in the general discussion.

Paralleling younger adults, there was no evidence that semantic associations between words and auditory contexts were being used in older adults.

## General Discussion

We aimed to determine whether the positive effects of background music on episodic memory that have been reported for both younger and older adults (Ferreri et al., 2013, 2014, 2015; Bottiroli et al., 2014) are observed when music is stopped before task performance (music as neuropsychological priming, or Mozart effect), and whether preferred music has an advantage over non-preferred. We predicted that music would show strong positive effects due to its non-distractive presence (not working as an interfering background), and that preferred music would have an advantage due to its role in maximizing reward. We ran our main study on a sample of 51 younger adults, and we complemented our approach with a pilot study on 12 older participants, divided into low- and high-functioning according to their performance on the Mini Mental-State Examination test. We predicted that low-functioning older participants would show increased sensitivity to positive music effects compared to healthy younger and older adults.

Unlike our predictions, the main study on younger adults (Experiment 1) showed null advantages of music over silence or environmental sounds, and no advantages of preferred music over non-preferred. This applied to both item and source memory. Null results were supported by the outcomes of Bayesian analyses. In addition, a sensitivity power analysis carried out with G^∗^Power (Faul et al., 2007) showed that the size of our younger adults sample (*n* = 51) would reliably detect small effect sizes for general auditory context (η^2^p = 0.031) and preference-related auditory context (η^2^p = 0.026). Therefore, evidence for lack of effects on item or source memory seems solid in this group. Contrasting these null effects of prior-to-task music with the positive effects of background music that have been seen before (Ferreri et al., 2013, 2014, 2015; Bottiroli et al., 2014) suggests that eliminating the distraction caused by background music (Cassidy and MacDonald, 2007; Kämpfe et al., 2011; Thompson et al., 2012; Yang et al., 2015) may yield other losses. Specifically, it is possible that, for episodic memory, the arousal, mood or reward effects afforded by music backgrounds (Blood and Zatorre, 2001; Schellenberg, 2005; Salimpoor et al., 2013; Ferreri and Verga, 2016) are lost or attenuated when music is stopped before the task begins. Given that preference also had null effects, and preference is strongly linked to reward, it is possible that reward may be the key loss: music-related reward may no longer favor episodic memory if music is stopped before the task begins. In contrast, arousal may not have been totally irrelevant to performance: although heavy metal music (potentially arousing at the physiological level) did not outperform non-musical auditory contexts, it differed significantly from jazz (a less arousing genre) concerning effects on source memory. Determining the roles of music-related reward vs. physiological arousal as prior-to-task enhancers of episodic memory remains, thus, a challenge for future research.

Alternative explanations for our null results based on sample characteristics do not seem likely: it is known that musical expertise attenuates or eliminates the Mozart effect (Twomey and Esgate, 2002), but our participants were not musicians; it is also known that men are less sensitive to this effect than women (Gilleta et al., 2003), but the majority of our participants was female. One alternative explanation that may deserve future approaches relates to the short exposure time to auditory contexts we used in our study. Typically, the Mozart effect has been implemented with a few minutes of musical stimulation, while we used only 20 s. Our intention was to maximize the similarity with Ferreri et al.’s (2015) study on background music to optimize the comparison between background and prior-to-task music, but it is possible that our choice weakened the effects of priming auditory contexts. Future studies could work on this exposure time variable to shed light on this matter.

Our pilot study with low- vs. high-functioning older adults was less powered than we could wish and its results should be taken with caution: for effects of general auditory context, G^∗^power sensitivity analyses showed that our study could reliably detect medium main effects of auditory context (our effects were small – η^2^p = 0.04 and.03 for item and source memory, respectively), large effects of cognitive functioning (our effect on item memory was large, η^2^p = 0.26 but medium on source memory, η^2^p = 0.24), and large interactive effects between auditory context and cognitive functioning (our effect on item memory was large, η^2^p = 0.40 and medium for source memory, η^2^p = 0.09). For preference-related auditory context, we could reliably detect only large main effects of auditory context and the observed effects were small - η^2^p = 0.04 and.03 for item and source memory, respectively. The interaction between preference-related auditory context and cognitive functioning could be reliably detected also only under large effect sizes. We found a large effect size for item memory (η^2^p = 0.25), but a small one (η^2^p = 0.05) for source memory. Admitting that low power did not make us miss small, yet real effects, our findings point to a deviant pattern in low-functioning older adults: Unlike younger and older high-functioning adults, music had an advantage over silence in item memory, and non-preferred, rather than preferred music, carried this advantage. In terms of specific music genres, fado and hip-hip (the least preferred) outperformed the silence condition, while local traditional music did not. A striking aspect in low-functioning older participants was their very low performance under silence (see Figures 3–5). Finally, the advantage that ordering auditory contexts as silence, environmental sounds and music had on the source memory of healthy younger and older adults was not obvious in the low-functioning group.

The advantage of music over silence only in low-functioning older adults may have been due to recruitment of music-induced compensatory mechanisms (Ferreri and Verga, 2016) that were not activated in younger and healthy older adults. It may also have been related to the presence of extremely high - hence detrimental - levels of task-related arousal (Thompson et al., 2001) in low-functioning older adults compared to the other groups: anxiety may have been mitigated by music (Irish et al., 2006), highlighting the beneficial effects of music in low-functioning, but not in other adults. The possibility that mitigating anxiety was a key mechanism in enhancing performance would be consistent with low-functioning participants’ very low performance under silence (potentially anxiogenic), and their facilitated performance under the two least physiologically arousing music pieces (fado and hip-hop, with softer timbres compared to local traditional music) – which coincided with the least preferred genres. From this viewpoint, relaxation rather than lack of preference would be the key to the positive impact of music. Future studies adding anxiety measures before and after music listening may shed light on this matter. Concerning the findings that low-functioning participants did not commit the same error as the other groups in source memory - responding to memory for temporal order (recency memory, which item was seen most recently?) based on a logical order of sources, instead of responding to the specific question we asked them (in which auditory context?, see section “Discussion” on Experiment 1), this may point to emerging problems with recency memory in this population.

Low-functioning older participants shared some behaviors with the other two groups. First, primacy effects on item memory were observed in younger, but not in older adults – low- or high-functioning. From a methodological viewpoint, this means that the use of multiple experiment versions may be critical when studying younger adults, and the difference between low- and high-functioning older participants may be irrelevant in this respect. In addition, we saw no evidence that semantic associations between words and auditory contexts were being used in any of the groups. This may indicate that prior-to-task music is not submitted to semantic processing, or, more likely, that our stimulus materials provided little or no opportunities for semantic associations. Future experiments manipulating the amount of semantic associations between music and word lists could clarify this matter.

The main contribution of our study was to highlight the null impact of prior-to-task music on the episodic memory of healthy younger adults. Unlike extant research using background, task-concurrent music, we saw no advantage from music over silence or environmental sounds in a priming paradigm where music was stopped before the task began, possibly because music-related reward loses strength under these circumstances. Although our findings require future validation with direct comparisons between background vs. prior-to-task music effects on episodic memory, they contribute to raise the awareness that background and prior-to-task music effects may engage different mechanisms (see also Lake and Goldstein, 2011) and they should not be approached interchangeably.

As for our pilot study contrasting low- with high-functioning older adults, it helped raise a number of hypothesis – namely the possible role of music in low-functioning participants’ anxiety reduction in a prior-to-task music stimulation scenario, but future research using large samples is mandatory before conclusions can be drawn.

## Data Availability Statement

All datasets generated for this study are included in the article/Supplementary Material.

## Ethics Statement

Ethical review and approval was not required for Experiment 1 in accordance with the local legislation and institutional requirements. Experiment 2 was reviewed and approved by Instituto da Segurança Social da Madeira. The patients/participants provided their written informed consent to participate in these studies.

## Author Contributions

SS, FB, and SC conceptualized the study. SS and FB created the stimulus set, analyzed the data, and wrote the first draft of the manuscript. FB collected the data. SC revised the manuscript. All authors contributed to the article and approved the submitted version.

## Conflict of Interest

The authors declare that the research was conducted in the absence of any commercial or financial relationships that could be construed as a potential conflict of interest.

## Footnotes

1
https://www.youtube.com/watch?v=8myYyMg1fFE

2
https://www.neurobs.com/

## Supplementary Material

Supplementary Table 1 Appendices **A–D**.

Supplementary Table 2 Database older adults.

Spplementary Table 3 Database younger adults.
