# Corrigendum: The Mozart effect on the episodic memory of healthy adults is null, but low-functioning older adults may be an exception

**DOI:** 10.3389/fpsyg.2023.1225558

**Published:** 2023-06-13

**Authors:** Susana Silva, Filipa Belim, São Luís Castro

**Affiliations:** Center for Psychology at University of Porto, Faculty of Psychology and Education Sciences, University of Porto, Porto, Portugal

**Keywords:** music listening, Mozart effect, episodic memory, preference, aging

In the published article, there was an error in the **Funding** statement. The statement was incorrectly written as, “This work was funded by the Portuguese Foundation for Science and Technology (CPUP UID/PSI/00050/2013; FEDER/COMPETE2020 POCI-01-0145-FEDER-007294).” The correct **Funding** statement appears below.

