# Supplementary material for: The Mozart Effect on the Episodic Memory of Healthy Adults Is Null, but Low-Functioning Older Adults May Be an Exception
Source: Front Psychol. 2020 Nov 3;11:538194. doi: 10.3389/fpsyg.2020.538194 (PMC7670071; doi:10.3389/fpsyg.2020.538194)
Supplement: Supplementary Table 1 — Appendices A–D. [file Table_1.docx]

Supplementary Material

# Appendix A

Verbal stimuli used in Experiment 1 (Young-adult participants)

| Old words (n = 45) | | | |  | New words (n = 45) | | | |
| --- | --- | --- | --- | --- | --- | --- | --- | --- |
| Word | Length | Frequency | Status |  | Word | Length | Frequency | Status |
| 1) Declarar | 8 | 39 | Verb |  | Candeio | 7 | 39 | Noun |
| 2) Desligar | 8 | 39 | Verb |  | Escocês | 7 | 40 | Noun |
| 3) Carência | 8 | 40 | Noun |  | Ilícito | 7 | 40 | adjective |
| 4) Elementar | 8 | 40 | Adjective |  | Abjecção | 8 | 41 | Noun |
| 5) Provedor | 8 | 38 | Noun |  | Ciclone | 7 | 41 | Noun |
| 6) Religião | 8 | 40 | Noun |  | Grevista | 8 | 41 | Noun |
| 7) Alumínio | 8 | 41 | Noun |  | Impalpável | 10 | 41 | adjective |
| 8) Suculento | 9 | 41 | Adjective |  | Oitenta | 7 | 41 | Noun |
| 9) Embebedar | 9 | 43 | Verb |  | Subverter | 9 | 41 | Verb |
| 10) Totalizar | 9 | 43 | Verb |  | Congelador | 10 | 42 | Noun |
| 11) Subjugar | 8 | 44 | Verb |  | Entulho | 7 | 42 | Noun |
| 12) Enrascar | 8 | 45 | Verb |  | Descida | 7 | 43 | Noun |
| 13) Península | 9 | 45 | Noun |  | Comadre | 7 | 44 | Noun |
| 14) Reflexão | 8 | 45 | Noun |  | Contaminar | 10 | 44 | Verb |
| 15) Mostarda | 8 | 46 | Noun |  | Delator | 7 | 44 | Noun |
| 16) Restituir | 9 | 46 | Verb |  | Retirar | 7 | 44 | Verb |
| 17) Silveira | 8 | 46 | Noun |  | Manifestar | 10 | 45 | Verb |
| 18) Motivação | 9 | 47 | Noun |  | Relance | 7 | 45 | Noun |
| 19) Paliativo | 9 | 47 | Adjective |  | Entrada | 7 | 46 | Noun |
| 20) Conceder | 8 | 48 | Verb |  | Flutuar | 7 | 46 | Verb |
| 21) Autocrata | 9 | 49 | Noun |  | Cegonha | 7 | 47 | Noun |
| 22) Escavação | 9 | 49 | Noun |  | Cognome | 7 | 48 | Noun |
| 23) Emagrecer | 9 | 50 | Verb |  | Psiquiatra | 10 | 48 | Noun |
| 24) Prontidão | 9 | 50 | Noun |  | Replica | 7 | 48 | Noun |
| 25) Alcovitar | 9 | 51 | Verb |  | Urgente | 7 | 48 | adjective |
| 26) Capacete | 9 | 51 | Noun |  | Producente | 10 | 50 | adjective |
| 27) Documento | 9 | 51 | Noun |  | Condimento | 10 | 52 | Noun |
| 28) Revestir | 8 | 51 | Verb |  | Participar | 10 | 52 | Verb |
| 29) Infringir | 9 | 52 | Verb |  | Desempatar | 10 | 53 | Verb |
| 30) Prodígio | 8 | 52 | Noun |  | Bilhete | 7 | 54 | Noun |
| 31) Vasculhar | 9 | 53 | Verb |  | Mercado | 7 | 54 | Noun |
| 32) Arrecadar | 9 | 54 | Verb |  | Petrificar | 10 | 56 | Verb |
| 33) Retirado | 8 | 54 | Noun |  | Viscoso | 7 | 56 | Noun |
| 34) Capelinha | 9 | 55 | Noun |  | Descrédito | 10 | 57 | Noun |
| 35) Depilação | 9 | 55 | Noun |  | Feiticeira | 10 | 58 | Noun |
| 36) Explícito | 9 | 55 | Adjective |  | Particular | 10 | 58 | adjective |
| 37) Acetinar | 8 | 56 | Verb |  | Rotunda | 7 | 58 | Noun |
| 38) Denuncia | 8 | 56 | Noun |  | Pousada | 7 | 59 | Noun |
| 39) Repudiar | 8 | 56 | Verb |  | Afastado | 8 | 60 | adjective |
| 40) Imunidade | 9 | 57 | Noun |  | Colossal | 8 | 61 | Noun |
| 41) Palpitar | 8 | 57 | Verb |  | Parteira | 8 | 61 | Noun |
| 42) Cavalaria | 9 | 58 | Noun |  | Atenuante | 9 | 62 | Noun |
| 43) Segmentar | 9 | 58 | Verb |  | Produzir | 8 | 62 | Verb |
| 44) Benjamim | 8 | 59 | Noun |  | Reprovar | 8 | 62 | Verb |
| 45) Combinar | 8 | 60 | Verb |  | Tarifar | 7 | 62 | Verb |
| *M + SD* | 8.5 + 0.5 | 49.2 + 6.2 |  |  |  | 8.1 + 1.3 | 49.7 + 7.7 |  |
| *Verb, Noun, Adjective* |  |  | 19,22,4 |  |  |  |  | 11,18,6 |

# Appendix B

Table B1: Pool of music stimuli used in the pre-test made with 20 young adults (selected stimuli marked on the last column). Table B2: preference ratings and preference contrasts for the selected pieces.

Table B1. Pre-test stimuli

| ID | Performer – Piece  [Youtube link – timing of selection] | Genre | Inclusion in final test |
| --- | --- | --- | --- |
| **1** | **John 5 and The Creatures - HERE'S TO THE CRAZY ONES**  [<https://www.youtube.com/watch?v=Ov6t1yqrx7Y> 1’48’’- 2’08’’] | **Metal** | **selected** |
| 2 | Deftones - U, U, D, D, L, R, L, R, A, B, Select, Start | Metal |  |
| 3 | Metallica – Orion | Metal |  |
| 4 | J Dilla – Life | Hip Hop |  |
| 5 | Jay-Z - Dead Presidents | Hip Hop |  |
| 6 | Mobb Deep - Shook Ones (instrumental) | Hip Hop |  |
| **7** | **Robert Miles – Children**  **[**<https://www.youtube.com/watch?v=6QEPrDBMqJ0> 35’’- 55’’] | **Electronic** | **selected** |
| 8 | BICEP \| GLUE | Electronic |  |
| 9 | Popof - Do You Want Me | Electronic |  |
| **10** | **Thelonious Monk - Blue Monk**  [<https://www.youtube.com/watch?v=_40V2lcxM7k> 24’’-44’’] | **Jazz** | **selected** |
| 11 | Dave Brubeck - Take Five | Jazz |  |
| 12 | Open Source Trio – Altitude | Jazz |  |

Table B2. Preference ratings and contrasts for selected stimuli

|  | Piece 1 | Piece 7 | Piece 10 | Piece 1- 7 | Piece 1-10 | Piece 7-10 |
| --- | --- | --- | --- | --- | --- | --- |
| p01 | 5 | 5 | 8 | 0 | 3 | 3 |
| p02 | 4 | 7 | 7 | 3 | 3 | 0 |
| p03 | 7 | 8 | 3 | 1 | 4 | 5 |
| p04 | 7 | 9 | 7 | 2 | 0 | 2 |
| p05 | 9 | 9 | 3 | 0 | 6 | 6 |
| p06 | 7 | 5 | 4 | 2 | 3 | 1 |
| p07 | 6 | 5 | 4 | 1 | 2 | 1 |
| p08 | 7 | 6 | 8 | 1 | 1 | 2 |
| p09 | 7 | 5 | 6 | 2 | 1 | 1 |
| p10 | 4 | 7 | 6 | 3 | 2 | 1 |
| p11 | 7 | 10 | 8 | 3 | 1 | 2 |
| p12 | 8 | 3 | 4 | 5 | 4 | 1 |
| p13 | 8 | 8 | 7 | 0 | 1 | 1 |
| p14 | 7 | 3 | 7 | 4 | 0 | 4 |
| p15 | 3 | 6 | 7 | 3 | 4 | 1 |
| p16 | 8 | 3 | 7 | 5 | 1 | 4 |
| p17 | 3 | 2 | 4 | 1 | 1 | 2 |
| p18 | 3 | 4 | 3 | 1 | 0 | 1 |
| p19 | 9 | 4 | 2 | 5 | 7 | 2 |
| p20 | 3 | 9 | 2 | 6 | 1 | 7 |
| Mean | 6,1 | 5,9 | 5,35 | 2,4 | 2,25 | 2,35 |

# Appendix C

Verbal stimuli used in Experiment 2 (elderly participants)

| Old words (n = 10) | | | |  | New words (n = 10) | | | |
| --- | --- | --- | --- | --- | --- | --- | --- | --- |
| Word | Length | Frequency | Status |  | Word | Length | Frequency | Status |
| Declarar | 8 | 39 | Verb |  | Candeio | 7 | 39 | Noun |
| Desligar | 8 | 39 | Verb |  | Escocês | 7 | 40 | Noun |
| Carência | 8 | 40 | Noun |  | Ilícito | 7 | 40 | Noun |
| Elementar | 8 | 40 | Noun |  | Abjecção | 8 | 41 | Noun |
| Provedor | 8 | 38 | Noun |  | Ciclone | 7 | 41 | Noun |
| Religião | 8 | 40 | Noun |  | Grevista | 8 | 41 | Noun |
| Alumínio | 8 | 41 | Noun |  | Impalpável | 10 | 41 | Adjective |
| Suculento | 9 | 41 | Adjective |  | Oitenta | 7 | 41 | Noun |
| Embebedar | 9 | 43 | Verb |  | Subverter | 9 | 41 | Verb |
| Totalizar | 9 | 43 | Verb |  | Congelador | 10 | 42 | Noun |
| *M + SD* | 8.3 + 0.5 | 40.4 + 1,6 |  |  |  | 8.0 + 1.2 | 40.7 + 0.8 |  |
| *Verb, Noun, Adjective* |  |  | 4,5,1 |  |  |  |  | 1,8,1 |

# Appendix D

Table D1. Mean accuracy rates per stimulus

|  | Silence | Environmental  sounds | Music 1 | Music 2 | Music 3 |
| --- | --- | --- | --- | --- | --- |
| Younger adults |  |  |  |  |  |
| Item memory | 0.690 | 0.679 | 0.751 | 0.740 | 0.718 |
| Source memory | 0.257 | 0.294 | 0.505 | 0.400 | 0.307 |
| Older adults |  |  |  |  |  |
| Item memory | 0.583 | 0.583 | 0.833 | 0.708 | 0.541 |
| Source memory | 0.291 | 0.291 | 0.541 | 0.458 | 0.291 |

Table D2. Mean d-prime values per stimulus

|  | Silence | Environmental  sounds | Music 1 | Music 2 | Music 3 |
| --- | --- | --- | --- | --- | --- |
| Younger adults |  |  |  |  |  |
| Item memory | 2.020 | 1.910 | 2.139 | 2.315 | 2.044 |
| Source memory | 0.537 | 0.712 | 1.206 | 0.864 | 0.360 |
| Older adults |  |  |  |  |  |
| Item memory | 3.426 | 3.381 | 5.240 | 4.310 | 3.071 |
| Source memory | 1.731 | 0.866 | 1.897 | 1.277 | 0.037 |
